# Identification of heat shock protein family A member 5 (HSPA5) targets involved in nonalcoholic fatty liver disease

**DOI:** 10.1038/s41435-023-00205-y

**Published:** 2023-05-08

**Authors:** Aliya Rehati, Buzukela Abuduaini, Zhao Liang, Dong Chen, Fangping He

**Affiliations:** 1 Department of Gastroenterology, The First Affiliated Hospital of Xinjiang Medical University, Urumqi, 830054 Xinjiang China; 2 Department of Intensive Care Unit, The First Affiliated Hospital of Xinjiang Medical University, 393 South Li Yu Shan Road, Urumqi, 830054 Xinjiang China; 3 Department of General Surgery, The First Affiliated Hospital of Xinjiang Medical University, Urumqi, 830054 Xinjiang China; 4 ABLife BioBigData Institute, Wuhan, 430075 Hubei China

**Keywords:** Genetics, RNA splicing

## Abstract

Heat shock protein family A (Hsp70) member 5 (HSPA5) is an endoplasmic reticulum chaperone, which regulates cell metabolism, particularly lipid metabolism. While HSPA5’s role in regulating cell function is well described, HSPA5 binding to RNA and its biological function in nonalcoholic fatty liver disease (NAFLD) is still lacking. In the present study, the ability of HSPA5 to modulate alternative splicing (AS) of cellular genes was assessed using Real-Time PCR on 89 NAFLD-associated genes. RNA immunoprecipitation coupled to RNA sequencing (RIP-Seq) assays were also performed to identify cellular mRNAs bound by HSPA5. We obtained the HSPA5-bound RNA profile in HeLa cells and peak calling analysis revealed that HSPA5 binds to coding genes and lncRNAs. Moreover, RIP-Seq assays demonstrated that HSPA5 immunoprecipitates specific cellular mRNAs such as *EGFR, NEAT1, LRP1* and *TGFß1*, which are important in the pathology of NAFLD. Finally, HSPA5 binding sites may be associated with splicing sites. We used the HOMER algorithm to search for motifs enriched in coding sequence (CDs) peaks, which identified over-representation of the AGAG motif in both sets of immunoprecipitated peaks. HSPA5 regulated genes at the 5′UTR alternative splicing and introns and in an AG-rich sequence-dependent manner. We propose that the HSPA5-AGAG interaction might play an important role in regulating alternative splicing of NAFLD-related genes. This report is the first to demonstrate that HSPA5 regulated pre-RNA alternative splicing, stability, or translation and affected target protein(s) via binding to lncRNA and mRNA linked to NAFLD.

## Introduction

Nonalcoholic fatty liver disease (NAFLD) is associated with an increased risk of cirrhosis and liver-related deaths [1]. NAFLD is characterized by the accumulation of excess liver triacylglycerol, inflammation and liver damage [2]. Hepatic inflammation in fatty livers involves resident and recruited macrophages with expressed toll like receptors (TLRs) and inflammasomes, which respond to damage associated molecular patterns (DAMPs) released from dead hepatocytes. Hepatic steatosis and fibrosis in NAFLD are driven by the activation of hepatic stellate cells expressing TLRs into collagen-producing myofibroblasts, which can induce a vicious cycle of inflammation and further fibrosis [3]. Previous reports indicate that the immune/inflammatory response plays an important role in the development and progression of NAFLD [4]. However, the contribution of the immune system and fibrosis to NAFLD is still under investigation.

The Heat shock protein family A (Hsp70) member 5 (HSPA5) is also known as GRP78 [5]. The HSPA5 gene encodes the binding immunoglobulin protein (BiP) and is localized in the Endoplasmic Reticulum (ER) lumen. “The ability of BiP to modify its function via post-translational modifications is one of its most essential functions” [6], BiP a regulator for Ca^2+^ homeostasis in the ER, as well as a facilitator of ER-associated protein degradation (ERAD) via retrograde transportation of aberrant proteins across the ER membrane [7]. BiP is also proposed to have an important function related to immunity. Interestingly, GRP78/HSPA5 is a universal therapeutic target for hepatocellular carcinoma and chronic liver disease [8]. For example, the CD5-like (CD5L) promotes liver cancer cell proliferation and anti-apoptotic responses by binding to HSPA5/GRP78 [9]. CD147 induces the unfolded protein response (UPR) to inhibit apoptosis and chemo-sensitivity by increasing Bip transcription in hepatocellular carcinoma [10]. Liver-specific loss of HSPA5/GRP78 perturbs the global unfolded protein response and exacerbates a spectrum of acute and chronic liver diseases [9].

HSPA5 was highly expressed in NAFLD and the levels of expression could be correlated to the disease state [11]. The global unfolded protein response is perturbed by liver-specific loss of GRP78, which in turn, is associated with more severe acute and chronic liver diseases [12]. An imbalance in lipid distribution to hepatocytes and metabolism causes hepatocyte steatosis. Vaspin is a novel adipokine that functions as a regulator of hepatocyte steatosis via the GRP78 receptor, effectively reducing hepatocyte fibrosis via activation of AMP-activated protein kinase (AMPK) and decreasing NF-κB gene expression [13]. However, how HSPA5 functions in NAFLD is not well understood.

In this study, we hypothesized that HSPA5 may play an important biological role through its RNA binding function. For example, HSPA5 could combine with pre-mRNA/mRNA transcribed from genes that encode for proteins playing a role in nonalcoholic fatty liver disease. The HSPA5-RNA interaction modulates gene expression, subsequently affecting the initiation and development of NAFLD. To test this hypothesis, we performed RNA-immunoprecipitation sequencing (RIP-Seq) on HSPA5 in HeLa cells. This work clearly defined a complex genome-wide HSPA5-RNA interaction map in a human cancer cell line and indicated that HSPA5 binds to the exon-intron boundary and regulates various alternative splicing (AS) events. These findings provide new knowledge on the HSPA5 regulation mechanism at the pre-mRNA splicing level and speculates on the novel functions of HSPA5 in multiple biological processes.

## Materials and methods

### Cloning and plasmid construction

Total RNA was extracted from the human cervical carcinoma (CC) cell line HeLa (CCTCC@GDC0009) obtained from China Center for Type Culture Collection, Wuhan, Hubei, China. Total RNA was reverse transcribed and the human *Hspa5* gene fragment was amplified using PCR. Primer pairs were designed with CE Design V1.04 (Vazyme Biotech Co., Ltd). The gene-specific primer sequences are listed in Table 1.

**Table 1:** RT-PCR primers for gene amplification.

Primer	Sequence (5’to3’)
HSPA5-Forward	5’ GGGAGGTGTCATGACCAAAC 3'
HSPA5-Reverse	5’ GCAGGAGGAATTCCAGTCAG 3'
GAPDH- Forward	5’ CGGAGTCAACGGATTTGGTCGTAT 3'
GAPDH- Reverse	5’ AGCCTTCTCCATGGTGGTGAAGAC 3'
LRP1-Forward-1	5' AAGAGCAGCGAGGAGTGAAGC 3'
LRP1-Revers-1	5' GTGAAGAGGTAGAGTTCCGAGCC 3'
LRP1-Forward-2	5' GTGGACGAGTTCCGCTGCAA 3'
LRP1-Revers-2	5' CCGAGCCATCCCCACAGTCA 3'
NEAT1-Forward	5' TTGCATAGCTGAGCGAGCCC 3'
NEAT1-Revers	5' TCCCTGCCTTCCTCCTTCCA 3'

The pIRES-hrGFP-1a vector (240031, Agilent Technologies) was digested with *Eco*RI (NEB, 3101S) and *Xho*I (NEB R0146V) at 37 °C for 2–3 h. Then enzyme-digested vector was run on a 1.0% agarose gel and purified with the Qiagen Column Kit (MinElute PCR Purification Kit, Qiagen, 28004). The DNA fragment corresponding to the full-length *Hspa5* cDNA was gel-purified using the MinElute PCR Purification Kit (Qiagen, 28004), and then cloned into the pIRES-hrGFP-1a vector using the hot fusion method with T4 DNA ligase (NEB, M0202V) and the ClonExpress® II One Step Cloning Kit (Vazyme, C112). The vector also harbors a FLAG tag fused to the 3’ end of APEX1. Ligated products were introduced into *Escherichia coli* DH5α by chemical transformation. Cells were plated onto LB agar plates containing 30 µg/ml ampicillin (Sigma, 7177-48-2), and incubated at 37 °C overnight. Colonies were screened by colony PCR (28 cycles) using universal primers that prime onto the vector backbone. The insert sequence was verified by Sanger sequencing.

### Cell culture and transfections

HeLa cells were cultured at 37 °C with 5% CO_2_ in Dulbecco’s Modified Eagle’s Medium (DMEM) with 10% fetal bovine serum (FBS), 100 μg/ml streptomycin and 100 U/ml penicillin. Transfection of HeLa cells with the recombinant plasmid was performed using Lipofectamine 2000 (Invitrogen, Carlsbad, CA, USA) according to the manufacturer’s protocol. Transfected cells were harvested after 48 h for real-time quantitative PCR (RT-qPCR) and western blot analysis.

### Assessment of gene expression

Total RNA from HeLa cells was converted into cDNA as noted above and RT-qPCR was performed on the Bio-Rad S1000 with the Bestar SYBR Green RT-PCR Master Mix (DBI Bioscience, Shanghai, China). The list of genes assessed and their corresponding primers are presented in Table 1. Glyceraldehyde-3-phosphate dehydrogenase (*Gapdh*) gene expression served as the control for assessing the effects of *Hspa5* overexpression. The concentration of each transcript was then normalized to *Gapdh* mRNA levels using the 2^−^^ΔΔCT^ method [14]. Statistical comparison was performed with the GraphPad Prism software (San Diego, CA) using the paired Student’s *t* test. Data were analyzed with the two-way analysis of variance (ANOVA).

### Immunoprecipitation

HeLa cells were first lysed on ice in ice-cold lysis buffer (1 × PBS, 0.5% sodium deoxycholate, 0.1% SDS, 0.5% NP40) with RNase inhibitor (TAKARA, 2313U) and a protease inhibitor (Solarbio, Cat. No. 329986) for 5 min. The mixture was then vortexed vigorously and centrifuged at 13,000 × *g* at 4 °C for 20 min to remove cell debris. The supernatant was incubated overnight with DynaBeads protein A/G (Thermo, Cat. No. 26162) conjugated with anti-flag antibody (Sigma, F1804) or control IgG-antibody (CST, 2797s). Low-salt wash buffer, high-salt wash buffer and 1X Polynucleotide Kinase Buffer were used to wash the beads with the respective antibodies. The beads were resuspended in Elution Buffer (50 mM Tris-Cl (pH = 8.0), 10 mM EDTA (pH = 8.0), 1% SDS at 70 °C for 20 min and divided into two groups, one for RNA isolation from HSPA5-RNA complexes and another for western blot analysis.

### Western blot

To elute the complex, the sample was placed in boiling water with 1X SDS sample buffer for 10 min. After that, the sample was separated on 10% SDS-PAGE and transferred to a membrane (Millipore, ISEQ00010). The membrane was blocked in TBST buffer (20 mM Tris-buffered saline and 0.1% Tween-20) containing 5% nonfat milk powder for 1 h at room temperature before incubating with the primary antibody, anti-Flag antibody (1:2000, Sigma, F7425) or anti-actin antibody (1:2000, CUSABIO), and then with HRP-conjugated secondary antibody (anti-mouse or anti-rabbit 1:10,000). (Abcam). Using the enhanced chemiluminescence reagent, secondary antibody was detected (Advansta, K-12045-D10).

### RIP-seq library preparation and sequencing

The HSPA5-bound RNAs were isolated using TRIzol (Invitrogen) after immunoprecipitation of anti-Flag-HeLa lysate. Complementary DNA (cDNA) libraries were prepared with the KAPA RNA Hyper Prep Kit (KAPA, Cat. No. KK8541) according the manufacturer’s procedure. Each of the IP and input groups libraries were prepared in replicates following the manufacturer’s instructions. A total of two RNA libraries were subjected to the Illumina Hi-Seq X Ten second-generation sequencing platform to obtain HSPA5-binding targets. Library preparation and RNA sequencing was repeated three times.

### Data analysis

Sequencing reads were mapped to the human GENCODE Release 23 genome (GRCh38.p3) using TopHat 2 and only uniquely mapped reads were used for the subsequent analyses. The genome-level binding sites for HSPA5 were identified using the “ABLIRC” method, a workflow for peak calling and analyzing CLIP-seq sequencing datasets (https://ablifedev.github.io/ABLIRC/). Peaks were formed from reads that had an overlap of at least one bp. Using computational simulation, sequence reads with the same lengths and counts as reads in the peaks were generated at random for each gene. After further mapping the generated reads to the corresponding genes, the overlapping reads were used to create random max peak height. This procedure was repeated over 500 iterations. All the observed peaks with heights greater than those of random maximum peaks (*p* < 0.05) were selected. The IP and input samples were independently analyzed via the simulation and the IP peaks that overlapped with Input peaks were removed. The HOMER software (http://homer.ucsd.edu/homer/motif/) was used to identify the IP target genes based on the peaks and IP protein binding motifs.

### Functional enrichment analysis

Using the KOBAS 2.0 server (http://kobas.cbi.pku.edu.cn/), KEGG pathways and Gene Ontology (GO) keywords were identified to classify the functional categories of peak linked genes (target genes). The enrichment of each term was determined using the Benjamini-Hochberg False Discovery Rate and Hypergeometric tests.

## Results

### RIP-seq analysis of HeLa cells revealed the global RNA binding characteristics for HSPA5

**Fig. 1:**
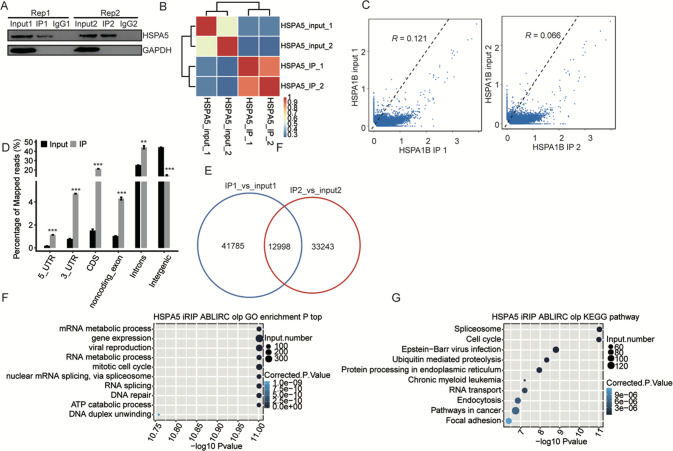
Transcriptome-wide identification of HSPA5 binding targets using RIP-seq. **A** Western blot analysis of HSPA5 immunoprecipitates using anti-Flag monoclonal antibody. Two replicates were performed. **B** Heat map clustering analysis of sample correlation based on the normalized mapped reads of each gene showing that two immunoprecipitated samples were clustered together. **C** Scatter plot showing Pearson correlation between immunoprecipitated and input samples. **D** Reads distribution across the reference genome. Error bars represent mean ± SEM. ***p < 0.001, **p < 0.01. **E** Venn diagram showing the overlap of HSPA5 binding peaks obtained from two replicates of iRIP-seq. The peaks were called with the ABLIRC algorithm. **F** The top 10 enriched GO biological processes of the HSPA5-bound genes. **G** The top 10 enriched KEGG pathways of the HSPA5-bound genes.

Western blot analysis confirmed the presence of HSPA5 protein in total cell lysate of HeLa cells and HSPA5 IP fraction, while no HSPA5 was detected in the IP fraction of the negative control IgG (Fig. 1A and Additional 1). The cDNA libraries of RNAs from anti-HSPA5 and IgG immune-precipitates were sequenced using the Hi-Seq X Ten platform, a total of 538,367 and 20,434,688 uniquely mapped reads from the anti-HSPA5 and IgG immune-precipitates, respectively, were recovered for further analysis (Additional file 2). The diagonal correlation matrix of the heat map (Fig. 1B and Additional 3) shows that the Pearson correlation between HSPA5 overexpression and control cells and the biological replicates was highly similar (Additional 4).

As shown in Fig. 1C, IP samples were significantly enriched compared with the input control. Collectively, these results reinforced the success of the RIP-seq experiments. Sequencing data obtained from HSPA5 overexpressed HeLa cells conjugated with the flag antibody demonstrated that the proportion of scattered reads in the IP group was higher than the control group, suggesting that HSPA5 binding sites may be associated with splicing sites (Additional 5). The group of control IP sample reads mapped to the reference genome were mainly distributed in intron and coding sequence (CDs) regions, while those in the IP group were in CDs, introns and UTR regions. Importantly, we observed that the fractions of clean reads that mapped to intergenic and intronic regions were significantly higher in HSPA5 immunoprecipitates than in IgG immunoprecipitates (Fig. 1D and Additional 6). The overlap of HSPA5 binding peaks between the replicate samples was high (Fig. 1E and Additional 7).

GO enrichment analysis was performed to further explore the biological function of differentially expressed genes (DEGs) using two different analytical strategies. The top biological process terms within the GO analysis that involved upregulation or downregulation of genes in the presence of overexpressed HSPA5 are presented in Fig. 1F, most of the terms overlapped with the top ten biological function terms which were RNA binding, protein serine kinase activity, helicase activity and transferase activity (Additional file 8). Most of the terms overlapped with the top 10 KEEG pathway including the mRNA processing, protein transport, cell cycle and mRNA splicing via spliceosome and presented in Fig. 1G and Additional file 9. Based on the KEGG analysis, the top ten pathways involved in up‐ and downregulated DEGs are presented in Fig. 1H and Additional file 10. As noted from the GO analyses, many pathways were associated with the immune-inflammatory response and proliferation while the focal adhesion pathway was significantly different. The above results showed that the top 10 paths of HSPA5 binding target gene enrichment in the two experiments were mainly coding genes and long non-coding RNAs (lncRNA). In the absence of any report on HSPA5 binding RNA, we speculate that HSPA5 may play a regulatory role by binding to the RNA of target genes. We focused on the top HSPA5 binding genes from the two experiments, which are NEAT1, LRP1, EGFR and TGFB1.

### Analysis of the HSPA5 binding motifs

**Fig. 2:**
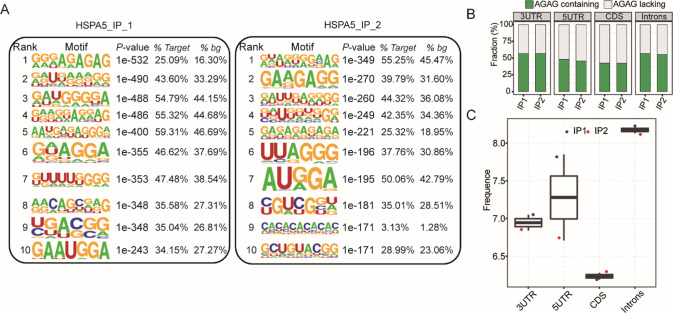
Analysis of the over-represented motifs indirectly bound by HSPA5. **A** The top 10 over-represented motifs in HSPA5 binding peaks located in the CDS regions. **B** Bar plots showing the fraction of the AGAG motif in the HSPA5 peaks located in different gene regions. **C** Box plots showing the frequency of the AGAG motif in the HSPA5 peaks located in different gene regions.

In the mRNA regions, HSPA5-peak read densities were higher closer to the transcription start sites (TSS) than those within the gene body regions and consistently high in the 3′ regions. We used the HOMER algorithm to search for motifs enriched in CDs peaks which identified over-representation of the AGAG motif in both sets of IP peaks (Fig. 2A). Given that the RIP approach captures the targets that are directly and indirectly bound by HSPA5, it is reasonable to assume that only a fraction of the HSPA5-bound target sites that we identified were directly bound by HSPA5 and contained the AGAG motifs. It could be possible that HSPA5 may prefer to directly bind some 5′UTR, and may bind to the other regions of a gene indirectly. To test this hypothesis, we analyzed the frequency of the AGAG motif in peaks located in different gene regions. Consistent with our hypothesis, the AGAG frequency in the 3′UTR peak was even higher than in the introns, which strongly suggests a direct role for HSPA5-AGAG interaction in regulating alternative splicing of NAFLD-related genes (Fig. 2B). The frequency of AGAG in the HSPA5 peaks located within the 5′UTR regions was significantly higher than those in the CDS and 3′UTR regions (Fig. 2C). Taken together, we have shown for the first time that the ABLIRC algorithm, which was successfully applied to extract the binding motifs of HSPA5 RNA binding protein, is useful to understand the regulatory role of protein-RNA interactions during gene expression of living cells.

**Fig. 3:**
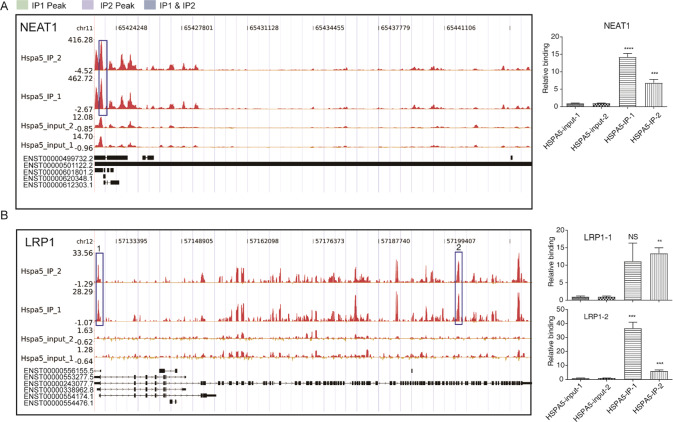
HSPA5 binds to genes involved in nonalcoholic fatty liver disease. Read density landscapes of HSPA5-binding peaks on transcripts of (**A**) NEAT1 and (**B**) LRP1.

### HSPA5 binds to genes involved in NAFLD

To further explore if HSPA5 binds to genes, we performed an iRIP-seq experiment in HeLa cells with a monoclonal antibody against HSPA5. Surprisingly, we noted that the NAFLD-related genes, including *NEAT1 and LRP1* (Fig. 3), were enriched in the HSPA5 immunoprecipitate. In addition, the top ten KEGG pathways of HSPA5 targets included Cell cycle, RNA transport and protein processing in endoplasmic reticulum and AMPK signaling pathway and the focal adhesion pathway (Fig. 1H). This results suggests that HSPA5 binds to genes involved in NAFLD pathology via regulation of alternative splicing, RNA stability, or its protein levels of NAFLD-associated genes.

## Discussion

Recent studies have revealed a new understanding of the ability of endoplasmic reticulum (ER) luminal chaperones like HSPA5/GRP78/BiP to escape to the cell surface in response to ER stress, where they control cell signaling, proliferation, apoptosis, and immunology [15]. GRP78 silencing increases the intracellular concentrations of essential polyunsaturated fats, furthermore, GRP78 inhibited both insulin-dependent and ER stress–dependent SREBP-1c proteolytic cleavage [16]. HSPA5 is an endoplasmic reticulum chaperone that regulates cell metabolism, in particular, lipid metabolism. However, it is also postulated that GRP78 may have RNA binding ability via interaction capture [17, 18]. In our study, we analyzed HSPA5 binding motifs peak calling and noted that HSPA5 regulated most genes via 5′UTR alternative splicing and introns and in a manner dependent on AG-rich sequences, where the HSPA5-AGAG interaction might play an important role in regulating alternative splicing of NAFLD-related genes. According to these findings, a G‐Rich element forms a G‐quadruplex and regulates the b-site APP cleaving enzyme 1 (BACE1) mRNA alternative splicing [19], suggesting that HSPA5-AGAG may be a new therapeutic target for liver-related diseases.

Interestingly, the HSPA5 target’s genes were enriched in the pathways related to the Cell cycle, RNA transport, and protein processing in the endoplasmic reticulum and AMPK signaling pathway, AMPK has been shown to have an important function in NAFLD. AMPK is regarded as the master regulator of several proteins involved in aging, inflammation, redox, and lipid and glucose metabolism [20]. Its activity causes the inhibition of de novo lipogenesis (DNL) via suppression of SREBP1c and ChREBP [21]. AMPK also phosphorylates ChREBP at the Ser568 position, inducing re-binding to 14-3-3 protein and subsequent inactivation, as well as inhibiting lipid synthesis [22, 23]. These results indicate that HSPA5 plays a critical role in cell viability and cell metabolism which play a critical role in NAFLD pathophysiology.

In this study, we focused on the top HSPA5 binding genes, *NEAT1, LRP1, EGFR*, and *TGFB1*. A previous study reported that the lncRNA of nuclear paraspeckle assembly transcript 1 (*NEAT1*) was upregulated in NAFLD [24], in addition, the down‐regulation of lncRNA‐NEAT1 alleviated NAFLD via the mTOR/S6K1-signaling pathway [14]. LncRNA-NEAT1 promotes hepatic lipid accumulation via regulation of miR-146a-5p/ROCK1 in NAFLD [24]. The lncRNA NEAT1-MicroRNA-140 axis exacerbates nonalcoholic fatty liver by interrupting AMPK/SREBP-1 signaling [25]. These results indicate that HSPA5 is involved in the development of NAFLD via regulation of NAFLD-related gene expression, transcription and alternative splicing.

The top HSPA5-binding proteins as well as low-density lipoprotein receptor-related protein-1 (LRP1) are involved in a number of cellular processes including intracellular signaling, lipid balance, and clearance of apoptotic cells [14]. A different study reported that LRP1 protects against hepatic insulin resistance and hepatic steatosis [26]. In addition, the epidermal growth factor receptor (EGFR), a member of the protein kinase superfamily, is involved in cell proliferation [27]. EGFR inhibition attenuates liver fibrosis and the development of hepatocellular carcinoma [28] while restoration of EGFR rescues fatty liver regeneration in mice [29]. Transforming growth factor-beta 1 (TGFβ1) is a secreted ligand of the transforming growth factor-beta (TGFβ) superfamily of proteins and is related to the regulation of programmed cell death, angiogenesis, and immune response [30]. A significant increase in the abundance of TGFβ1 mRNA in platelets is associated with nonalcoholic steatohepatitis (NASH) [31]. These results suggest that HSPA5 might regulate liver cellular processes via binding to NEAT1, LRP1, EGFR, and TGFB1, which may be important in the pathogenesis of NAFLD.

In this study, we established that HSPA5 binding is enriched toward genes encoding for splicing-associated factors as well as other genes encoding proteins with important roles in the development of NAFLD. We then demonstrated that HSPA5 binds to and impacts the splicing, transcription and translation of lncRNA and mRNA of its target genes which are associated with immune-inflammatory and metabolism functions. These new findings open up the potential of GRP78/HSPA5 as a suitable clinical therapeutic target to treat NAFLD. In conclusion, HSPA5 may play an important biological role through its RNA binding function. HSPA5 binding to the RNA transcripts of the NAFLD-associated genes affected mRNA alternative splicing, stability, and transcription, which impacted the encoded proteins. Taken together, this contributed to the pathophysiology of NAFLD, thus supporting the critical role of HSPA5 in NAFLD.

## Supplementary information

supplementary legends

Additional file1

Additional file2

Additional file3

Additional file4

Additional file5

Additional file6

Additional file7

Additional file8

Additional file9

Additional file10

## Data Availability

The data in this publication are available under the GEO Series accession number GSE169538.
