# Supplementary material for: Identification of heat shock protein family A member 5 (HSPA5) targets involved in nonalcoholic fatty liver disease
Source: Genes Immun. 2023 May 8;24(3):124–9. doi: 10.1038/s41435-023-00205-y (PMC10266971; doi:10.1038/s41435-023-00205-y)
Supplement: Supplementary file 1 — supplementary legends [file 41435_2023_205_MOESM1_ESM.docx]

**Supplementary legends**

Additional file 1: HSPA5_relative_exp

Additional file 2: Obtain_the_high_quality_clean_reads

Additional file 3: Sample_correlation

Additional file 4: expressed_gene_RPKM

Additional file 5: Length_distribution_across_Genomic_Regions

Additional file 6: Repeated_peaks_in_multiple_analysis

Additional file 7: peak_mapping_distribution

Additional file 8: HSPA5_iRIP_ABLIRC_olp_GO_enrichment_F_top

Additional file 9: HSPA5_iRIP_ABLIRC_olp_GO_enrichment_P_top

Additional file 10: HSPA5_iRIP_ABLIRC_olp_KEGG_pathway
